# Supplementary material for: Association between oral microbiome and seven types of cancers in East Asian population: a two-sample Mendelian randomization analysis
Source: Front Mol Biosci. 2023 Nov 21;10:1327893. doi: 10.3389/fmolb.2023.1327893 (PMC10702768; doi:10.3389/fmolb.2023.1327893)
Supplement: Supplementary file 1 [file DataSheet1.ZIP › Supplementary Materials/MR plots for tongue/tongue═╝/Prostate cancer/pheno.463_to_prostate cancer_forest.pdf]

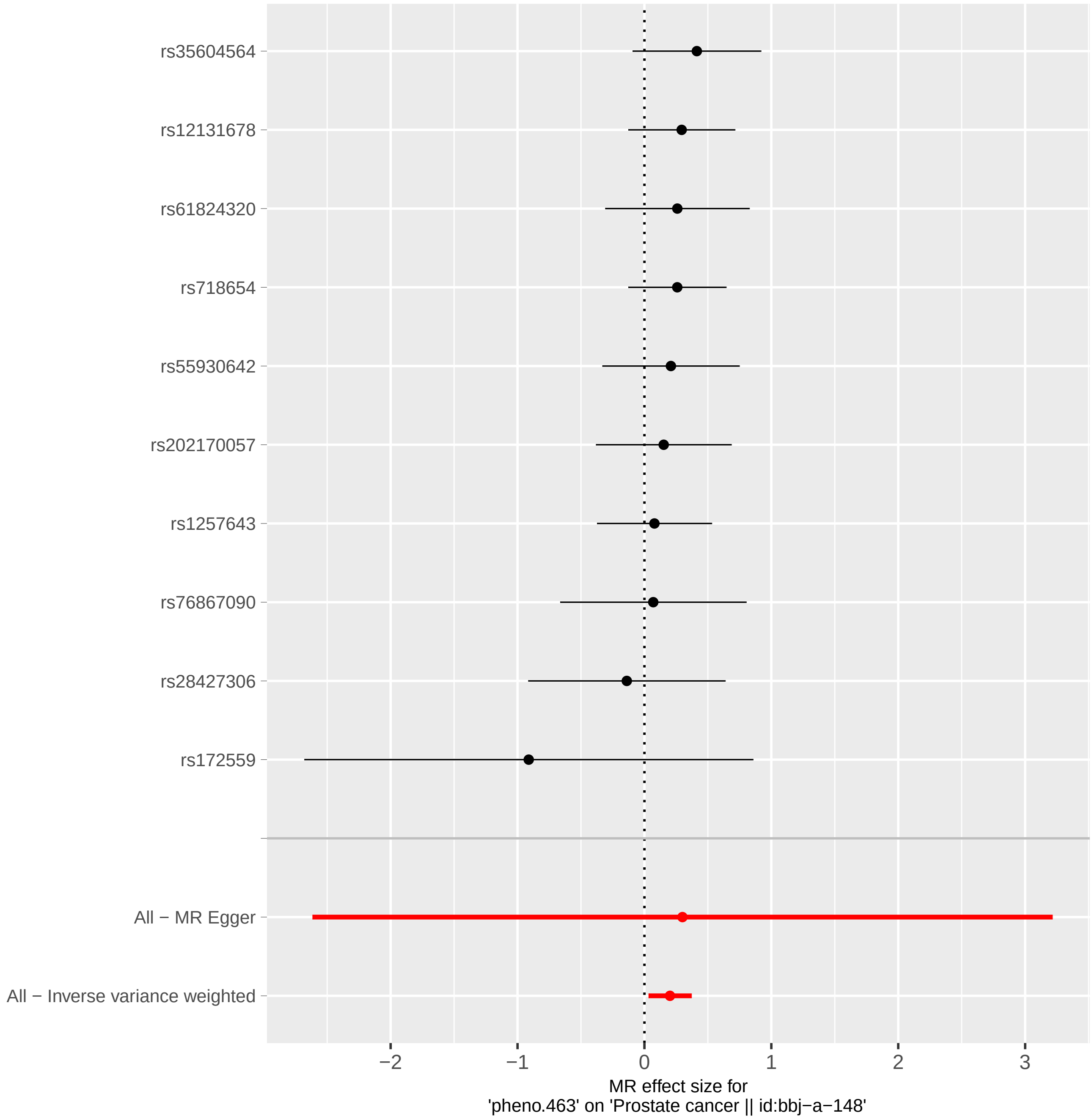

rs35604564
rs12131678
rs61824320
rs718654
rs55930642
rs202170057
rs1257643
rs76867090
rs28427306
rs172559
All − MR Egger
All − Inverse variance weighted
−2
−1
0
1
2
3
MR effect size for
'pheno.463' on 'Prostate cancer || id:bbj−a−148'
